# Metabolic engineering of microorganisms for L-alanine production

**DOI:** 10.1093/jimb/kuab057

**Published:** 2021-08-19

**Authors:** Pingping Liu, Hongtao Xu, Xueli Zhang

**Affiliations:** Tianjin Institute of Industrial Biotechnology, Chinese Academy of Sciences, Tianjin 300308, China; Key Laboratory of Systems Microbial Biotechnology, Chinese Academy of Sciences, Tianjin 300308, China; National Technology Innovation Center of Synthetic Biology, Tianjin 300308, China; Tianjin Institute of Industrial Biotechnology, Chinese Academy of Sciences, Tianjin 300308, China; Key Laboratory of Systems Microbial Biotechnology, Chinese Academy of Sciences, Tianjin 300308, China; National Technology Innovation Center of Synthetic Biology, Tianjin 300308, China; Tianjin Institute of Industrial Biotechnology, Chinese Academy of Sciences, Tianjin 300308, China; Key Laboratory of Systems Microbial Biotechnology, Chinese Academy of Sciences, Tianjin 300308, China; National Technology Innovation Center of Synthetic Biology, Tianjin 300308, China

**Keywords:** L-alanine, Metabolic engineering, Industrial production, Fermentation

## Abstract

L-alanine is extensively used in chemical, food, and medicine industries. Industrial production of L-alanine has been mainly based on the enzymatic process using petroleum-based L-aspartic acid as the substrate. L-alanine production from renewable biomass using microbial fermentation process is an alternative route. Many microorganisms can naturally produce L-alanine using aminotransferase or L-alanine dehydrogenase. However, production of L-alanine using the native strains has been limited due to their low yields and productivities. In this review, metabolic engineering of microorganisms for L-alanine production was summarized. Among them, the *Escherichia coli* strains developed by Dr. Lonnie Ingram's group which can produce L-alanine with anaerobic fermentation process had several advantages, especially having high L-alanine yield, and it was the first one that realized commercialization. L-alanine is also the first amino acid that could be industrially produced by anaerobic fermentation.

## Introduction

L-alanine is one of the smallest chiral compounds (Smith et al., 2006) and has been widely used in food, pharmaceutical, and veterinary fields for a long history. For example, it is used as a pre- and postoperative nutrition therapy together with other L-amino acids (Hols et al., 1999) in clinical medicine and used as sweetener for its sweet taste (Chibata et al., 1969; Lee et al., 2004) in food industry. Owing to the industrial and scientific development and the reduced cost, more applications of L-alanine are being successively exploited in recent years. A most attractive application is as one of the most important raw material for production of methylglycinediacetic acid (MGDA) (González et al., 2011), which is a newly synthetic green chelating agent with excellent performance and can be used in many types of cleaning products. The reduced L-alanine cost has accelerated MGDA development much more quickly. L-alanine also shows great potential for production of biodegradable and biocompatible polymers, such as polyesteramide, and engineered thermoplastics, such as polyamides and poly(amide-ester-imide)s (Bonillo Martínez et al., 2017; Mallakpour & Dinari, 2011; Zhou et al., 2016). The global annual demand of L-alanine is 50 000 tons today.

**Fig. 1:**
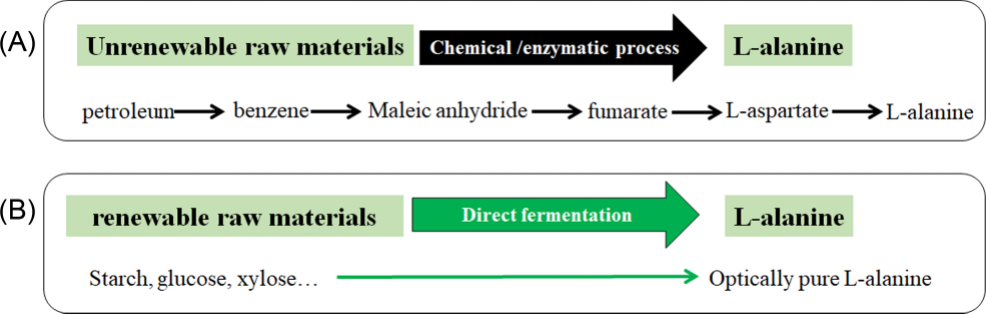
Two production routes for L-alanine production. (A) Enzymatic process using petroleum-based raw materials; (B) fermentation process using renewable biomass.

Traditionally, L-alanine was produced by enzymatic decarboxylation of L-aspartic acid using immobilized cells or cell suspensions of microorganisms owning an L-aspartic-β-decarboxylase as the biocatalyst (Chibata et al., 1965, 1984; Shibatani et al., 1979) in industrial production. This process has been proved to be highly efficient and the L-alanine yield can exceed 90% (Shibatani et al., 1979). L-aspartate is usually produced from fumarate by enzymatic catalysis using aspartate ammonia-lyase, whereas fumarate is primarily obtained from petroleum (Fig. 1A). The petroleum-based products have properties such as nonrenewability and environment unfriendliness, price dependence on global storage, fluctuations of petroleum prices, trade imbalances, and political considerations (Keasling, 2010). Alternately, an efficient fermentation process (Fig. 1B) for L-alanine production is considered to be feasible and especially advantageous from the industrial point of view, due to the advantages of using renewable and inexpensive sugars as the starting material (Katsumata & Hashimoto, 1996).

**Fig. 2:**
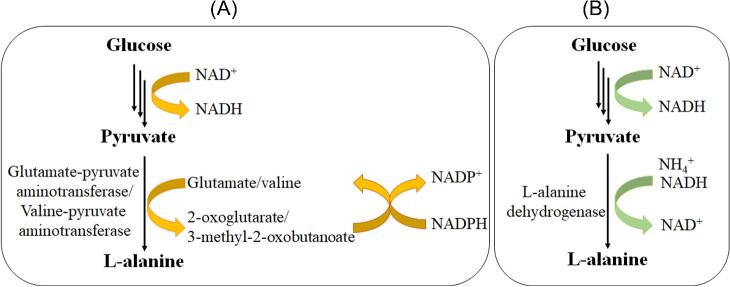
L-alanine synthesis in microorganisms by (A) aminotransferase and (B) L-alanine dehydrogenase.

Alanine is an essential component of cellular proteins and peptidoglycan. Production of L-alanine is regarded as an ancestral metabolism in the phylogenetic studies (Ravot et al., 1996). In most microorganisms, L-alanine is considered to be synthesized mainly for cell biosynthesis and synthesized by transamination (Fig. 2A) between pyruvate and glutamate or valine (Chibata et al., 1965; Hashimoto & Katsumata, 1998; Shibatani et al., 1979). However, strains using this route for L-alanine production have no practical use because the accumulation of L-alanine was not only in relatively low level but also in the form of DL-alanine (Hashimoto & Katsumata, 1993). Except the transaminase activity, researchers also find that some organisms possess L-alanine dehydrogenase (ALD) activity (Fig. 2B) for L-alanine synthesis (Hashimoto & Katsumata, 1998; Ohashima & Soda, 1979; Zhang et al., 2007). The nicotinamide adenine dinucleotide (NAD^+^)-dependent ALD can produce L-alanine from pyruvate and ammonia, such as in *Arthrobacter oxydans* (Hashimoto & Katsumata, 1993, 1998, 1999), *Bacillus sphaericus* (Ohashima & Soda, 1979), and *Clostridium* sp. P2. (Orlygsson et al., 1995). Yet, the slower fermentation rate and relatively lower yield of these strains prevent their application in industrial production. Fortunately, metabolic engineering has brought a ray of light. Nowadays, many microorganisms have been engineered for fermentative production of L-alanine, among which the engineered *Escherichia coli* strains developed by Dr. Lonnie Ingram's group realized commercialization. Currently, L-alanine produced by fermentation process accounts for more than 60% of the whole market. In this review, we will focus on metabolic engineering of microorganisms for L-alanine production. Furthermore, the industrial fermentative production of L-alanine will also be discussed.

## Production of L-alanine by Engineered Microorganisms Using Either Aerobic or Two-Stage Fermentation Process

Metabolic engineering of microorganisms for L-alanine production has been investigated since 1990s (Table 1). The native strain *A. oxydans* HAP-1 can produce large amounts of DL-alanine from glucose. By inactivation of the alanine racemase in this strain, a mutant strain DAN75 was obtained. The final L-alanine titer reached up to 75.6 g/l which was almost equal to the sum of D- and L-alanine produced by the parental strain. The optical purity of L-alanine was up to 97% (Hashimoto & Katsumata, 1998). This study demonstrated the possibility of L-alanine production using engineered strains. Yet, the fermentation time was too long (120 h) and the yield was only 0.16 g/g glucose.

**Table 1.** Summary of Alanine Producing Strains

Organisms	Modified properties	Fermentation conditions	Time (H)	Alanine (g/l)	Yield (g/g)	L-alanine optical purity (%)	References
*Zymomonas mobilis* CP4thi/pZY73	Transformant of *Z. mobilis* CP4thi with plasmid pZY73 expressing the *Bacillus sphaericus alaD*	Mineral salts medium, simple batch, 50 g/l glucose	26	8 g/l L-alanine	0.16	Not reported	Uhlenbusch et al. (1991)
*Escherichia coli* AL1	Transformant of *E. coli* JM105 with plasmid pOBP1 expressing the *Arthrobacter* sp. HAP1 *alaD*	Mineral medium, 20 g/l glucose, limited oxygen supply	40	8 g/l DL-alanine	0.41	Not reported	Katsumata and Hashimoto (1996)
*Corynebacterium glutamicum* AL107	Transformant of *C. glutamicum* ATCC 21 352 with plasmid pOBP107 expressing the *Arthrobacter* sp. HAP1 *alaD*	Mineral medium, corn steep liquor, 4 g/l DL-alanine, 200 g/l glucose, limited oxygen supply	70	71 g/l L-alanine	0.36	>99	Katsumata and Hashimoto (1996)
*Arthrobacter* sp. HAP-1	Wild alanine producing strain	Fed-batch, mineral medium, 150 g/l glucose	100	49.1 g/l L-alanine and 32.8 g/l D-alanine	0.55	Not reported	Hashimoto and Katsumata (1998)
*Arthrobacter* sp. DAN-75	*Arthrobacter* sp. HAP-1 deficient of alanine racemase	Fed-batch, mineral medium, 150 g/l glucose, 2 g/l D-alanine	120	75.6 g/l L-alanine and 1.2 g/l D-alanine	0.51	Not reported	Hashimoto and Katsumata (1998)
*Lactococcus lactis* NZ3950 (pNZ2650)	Transformant of *L. lactis* NZ3900 with plasmid pNZ2650 expressing the *B. sphaericus alaD*, △*ldhA, pepN*::*nisRK*,	K-NaPO_4_ buffer, 18 g/l glucose	17	13 g/l L-alanine	0.70	85–90	Hols et al. (1999)
*L. lactis* PH3950 (pNZ2650)	Transformant of *L. lactis* NZ3900 with plasmid pNZ2650 expressing the *B. sphaericus alaD*, △*ldhA, pepN*::*nisRK*, △*alr*	K-NaPO_4_ buffer, 18 g/l glucose, 0.2 g/l D-alanine	17	ND	ND	>99	Hols et al. (1999)
*E. coli* AL887 (pTrc99A-alaD)	Transformant of *E. coli* with pTrc99a-alaD expressing the *B. sphaericus alaD*, △*ldhA*, △*aceF*	Two-stage batch and feeding process, 50 g/l glucose, yeast extract	27	32 g/l L-alanine	0.63	Not reported	Lee et al. (2004)
*E. coli* ALS929(pTrc99A-alaD)	*E. coli* K-12, △*pfl*, △*pps*, △*aceEF*, △*poxB*, △*ldhA*, pTrc99a-alaD expressing the *B. sphaericus alaD*	Two-phase batch (aerobic cell growth and anaerobic alanine production) process	21.5	34 g/l L-alanine	0.86	Not reported	Smith et al. (2006)
*E. coli* ALS929(pTrc99A-alaD)	*E. coli* K-12, △*pfl*, △*pps*, △*aceEF*, △*poxB*, △*ldhA*, pTrc99a-alaD expressing the *B. sphaericus alaD*	Two-phase fed-batch (aerobic cell growth and anaerobic alanine production) process	48	88 g/l L-alanine	1	Not reported	Smith et al. (2006)
*E. coli* XZ132	*E. coli* W, △*pfl*, △*ackA*, △*adhE*, △*mgsA*, △*dadX*, △*ldhA*::*alaD* from *Geobacillus stearothermophilus*	Mineral medium, batch fermentation process, 120 g/l glucose	48	114 g/l L-alanine	0.95	>99	Zhang et al. (2007)
*C. glutamicum* △*ldhA*△*ppc* + alaD	Transformant of *C. glutamicum* with plasmid pCRD500 containing the *alaD* from *Lysinibacillus sphaericus*, △*ldhA*, △*ppc*	Oxygen deprivation (aerobic cell growth, harvested and washed, resuspended and alanine produced) batch, mineral salts medium	30	25 g/l L-alanine	0.74	Not reported	Jojima et al. (2010)
*C. glutamicum* △*ldhA*△*ppc* + *alaD* + *gapA*	Transformant of *C. glutamicum* with plasmid pCRD501 containing the *alaD* from *L. sphaericus* and *gapA*, △*ldhA*, △*ppc*	Oxygen deprivation (aerobic cell growth, harvested and washed, resuspended and alanine produced) batch, mineral salts medium	13	29 g/l L-alanine	0.86	>65.4	Jojima et al. (2010)
*C. glutamicum* △*ldhA*△*ppc*△*alr* + *alaD* + *gapA*	Transformant of *C. glutamicum* with plasmid pCRD501 containing the *alaD* from *L. sphaericus* and *gapA*, △*ldhA*, △*ppc*, △*alr*	Oxygen deprivation (aerobic cell growth, harvested and washed, resuspended and alanine produced) batch, mineral salts medium, 30 mM pyruvate	32	98 g/l L-alanine	0.83	>99.5	Jojima et al. (2010)
*E. coli* B0016-060BC	*E. coli* B0016, △*ldhA*, △*ackA-pta*, △*pflB*, △*adhE*, △*frdA*, △*dadX*:: *cI*^ts^857-*p*R-*p*L-*alaD*-FRT	Thermoregulated process, 33°C aerobic cell growth and 42°C oxygen-limited alanine production	40	120.8 g/l L-alanine	0.88	Not reported	Zhou et al. (2016)

L-ALD is the key for efficient L-alanine production. However, many microorganisms don't have this enzyme. Introducing L-ALD into the host strains has been commonly used for efficient L-alanine production. By overexpressing the *B. sphaericus* L-ALD *alaD* gene using plasmid pZY73, an engineered strain *Zymomonas mobilis* CP4thi was constructed. A two-stage fermentation procedure was carried out by growing cells in the first stage and washing and resuspending cells to produce L-alanine in the second stage. Low level of racemic alanine was produced during the anaerobic fermentation (Uhlenbusch et al., 1991). Hashimoto et al. also constructed two engineered strains for L-alanine production. The first one was *E. coli* AL1 which was a transformant of *E. coli* JM105 with plasmid-borne *alaD* from *Arthrobacter* sp. HAP1 and another one was *C. glutamicum* AL107 which was a transformant of *C. glutamicum* ATCC 21352 with plasmid expressing the same *alaD* of *Arthrobacter* sp. HAP1. When fermented under oxygen-limited conditions, *E. coli* AL1 produced 8 g/l DL-alanine with a yield of 0.41 g/g glucose whereas *C. glutamicum* AL107 produced 71 g/l L-alanine with a yield of 0.3 g/g glucose and a high optical purity of 99% (Katsumata & Hashimoto, 1996).

Another challenge for fermentative production of L-alanine is the formation of coproducts. To solve this problem, strains were designed by deletion of genes related to the competitive metabolic pathways. In an engineered L-alanine producing *Lactococcus lactis* strain, the native chromosomal lactate dehydrogenase gene *ldhA* and alanine racemase gene *alr* were both deleted and D-alanine was added to support cell growth (Hols et al., 1999). By deletion of both *aceF* and *ldhA* genes, an *E. coli* mutant AL887 was also obtained. The engineered strain *E. coli* AL887 (pTrc99A-alaD) could produce 32 g/l L-alanine with a yield of 0.63 g/g glucose using a two-stage batch fermentation process (Lee et al., 2004). *E. coli* mutant was further constructed by deletion of genes encoding pyruvate-formate lyase, phosphoenolpyruvate synthase, pyruvate oxidase, lactate dehydrogenase, components of the pyruvate dehydrogenase complex, and overexpression of *B. sphaericus alaD* gene. The obtained strain produced 88 g/l L-alanine with a yield approaching the theoretical maximum when a two-phase fed-batch procedure was used (Smith et al., 2006). A similar procedure was also used in *C. glutamicum*. The strain was genetically engineered for producing L-alanine by inactivating genes associated with organic acids production and overexpressing *Lysinibacillus sphaericus alaD* gene to direct carbon flux from organic acids to L-alanine (Jojima et al., 2010). The resulted strain produced 25 g/l alanine. Next, the glyceraldehyde 3-phosphate dehydrogenase was further overproduced to accelerate the glucose consumption and the titer of alanine increased to 29 g/l. After deletion of the *alr* gene encoding the native alanine racemase, the resulted strain produced 98 g/l L-alanine with a yield of 0.83 g/g glucose after 32 h fermentation under oxygen deprivation condition and adding 30 mM pyruvate (Jojima et al., 2010).

To efficiently produce L-alanine and reduce the L-alanine inhibition on cell growth, a thermoregulated genetic switch was designed in an engineered *E. coli* strain to dynamically control the expression of *Geobacillus stearothermophilus alaD* gene. Taking a fermentation process that an aerobic growth phase at 33°C with a 1 h thermoinduction at 42°C followed by an oxygen-limited phase at 42°C, the titer of L-alanine reached up to 120.8 g/l with a yield of 0.88 g/g glucose at the end of 42 h fermentation (Zhou et al., 2016).

## Production of L-alanine by Engineered *E. coli* Strains Using Anaerobic Fermentation Process

One important disadvantage of the aerobic or two-stage fermentation process is that lots of carbon sources are consumed for cell growth which would lead to relatively low yield and high production cost. Anaerobic fermentation process is better to obtain high yield since almost no carbon flux would go through TCA cycle and very few carbon source is used for cell growth whereas most carbon flux can go to designed product. However, since the cell mass is low, the specific productivity needs to be very high so that high titer and productivity can be realized. Dr. Lonnie Ingram's group developed a metabolic evolution strategy to solve this problem.

**Fig. 3:**
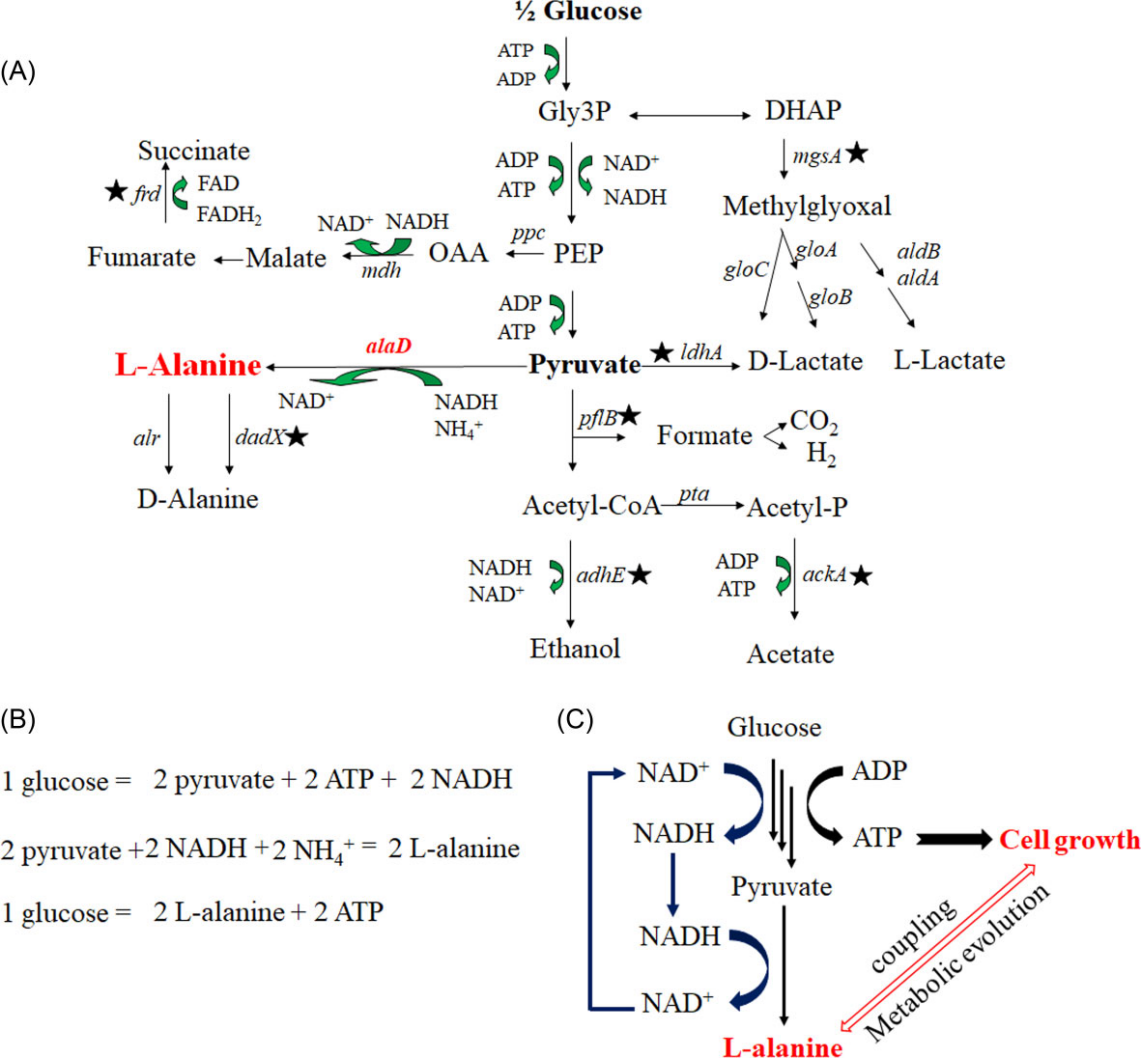
Alanine pathway in recombinant *E. coli* XZ132 (Zhang et al., 2007). (A) Native and recombinant fermentation pathways. The foreign gene, *G. stearothermophilus alaD*, is shown in *red. Solid stars* represent deletions of native genes in XZ132. Note that the native biosynthetic route for alanine production is omitted for simplicity. (B) L-alanine yield from 1 mol glucose for strain XZ 132. (C) Coupling of ATP production and growth to NADH oxidation and L-alanine production. Glucose is metabolized to pyruvate, ATP, and NADH. Energy conserved in ATP is utilized for growth and homeostasis, regenerating ADP. NADH is oxidized by alanine formation allowing glycolysis and ATP production to continue.

Starting from a lactate-producing strain SZ194, the native D-lactate dehydrogenase gene was replaced by the *alaD* gene from *G. stearothermophilus* (Zhang et al., 2007). In addition, the methylglyoxal synthase gene *mgsA* was deleted to improve cell growth and the major alanine recemase gene *dadX* gene was deleted to increase chiral purity. In the resulting strain, key enzymes related to the native mixed acid fermentation were all inactivated, and L-alanine became the only fermentative product (Fig. 3A). Under anaerobic conditions, synthesis of L-alanine was the only way to oxidize NADH which was produced during glycolysis. Continued L-alanine synthesis was required for continued glycolysis. Since all the energy required for cell growth were provided by glycolysis, L-alanine synthesis was thus coupled to cell growth (Fig. 3B, C). This provided a growth-based selection for improving L-alanine synthesis (Fig. 3). After metabolic evolution for 177 generations, an engineered strain XZ132 was obtained. This strain produced 114 g/l L-alanine with a yield of 0.95 g/g glucose in mineral medium. The chiral purity was greater than 99.5% (Zhang et al., 2007). In addition, all the foreign genes were integrated in the chromosome, thus no antibiotic was required to maintain plasmid. Expensive inducers were not needed since no inducible promoter was used during strain engineering. The strain was also adapted to mineral salts medium during the metabolic evolution. All these unique advantages of strain XZ132 were helpful for the industrial application.

## Commercial Production of L-alanine by Engineered *E. coli* Strains

**Fig. 4:**
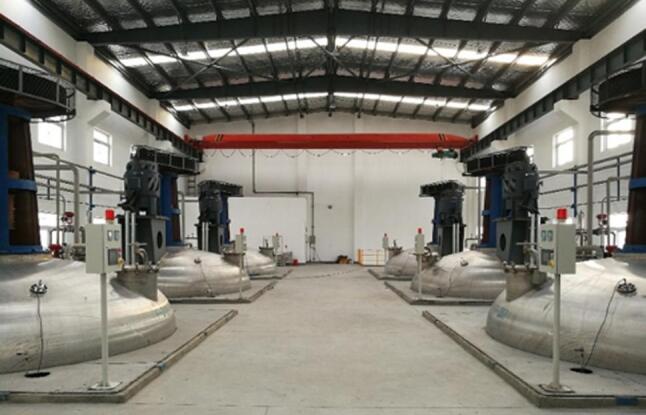
Industrial fermentative production of L-alanine.

The engineered *E. coli* strain XZ132 developed by Dr. Ingram's group was licensed to Anhui Huaheng Biotechnology Co., Ltd. (AHB), and commercial production of L-alanine by this strain and its derivatives has been realized since 2012. This is the first time to realize commercialization of fermentative production of L-alanine in the world. Now, two production lines for annual production of 23 000 tons L-alanine has been established by AHB. L-alanine can be successfully produced in 250 m^3^ fermentors (Fig. 4) and the yield was as high as in the 5 l fermentor. It should be noted that L-alanine is also the first amino acid that could be industrially produced by anaerobic fermentation.
